# A major new dimension in the problem of brain injury

**DOI:** 10.7554/eLife.72048

**Published:** 2021-08-10

**Authors:** Jonathan R Wolpaw, Jonathan S Carp

**Affiliations:** 1 National Center for Adaptive Neurotechnologies, Albany Stratton VA Medical Center Albany United States; 2 Albany Stratton VA Medical Center Albany United States

**Keywords:** neuroendocrine signaling, brain injury, neurohormones, postural asymmetry, nociceptive withdrawal reflex, left-right side, Rat

## Abstract

Evidence that neurohormones contribute to the contralateral effects of unilateral brain injury challenges a fundamental assumption of basic neuroscience and clinical neurology.

**Related research article** Lukoyanov N, Watanabe H, Carvalho LS, Kononenko O, Sarkisyan D, Zhang M, Andersen MS, Lukoyanova EA, Galatenko V, Tonevitsky A, Bazov I, Iakovleva T, Schouenborg J, Bakalkin G. 2021. Left-right side-specific endocrine signaling complements neural pathways to mediate acute asymmetric effects of brain injury. *eLife* **10**:e65247. doi: 10.7554/eLife.65247

As Hippocrates knew, an injury to one side of the brain, such as a stroke, affects muscle control on the opposite side of the body (Clarke and O’Malley, 1996). For centuries, it has been assumed that this contralateral effect is due entirely to abnormal activity in neural pathways from the injured side of the brain that cross the midline to activate the spinal cord neurons that control muscles on the opposite side of the body (Figure 1, left side).

**Figure 1.**
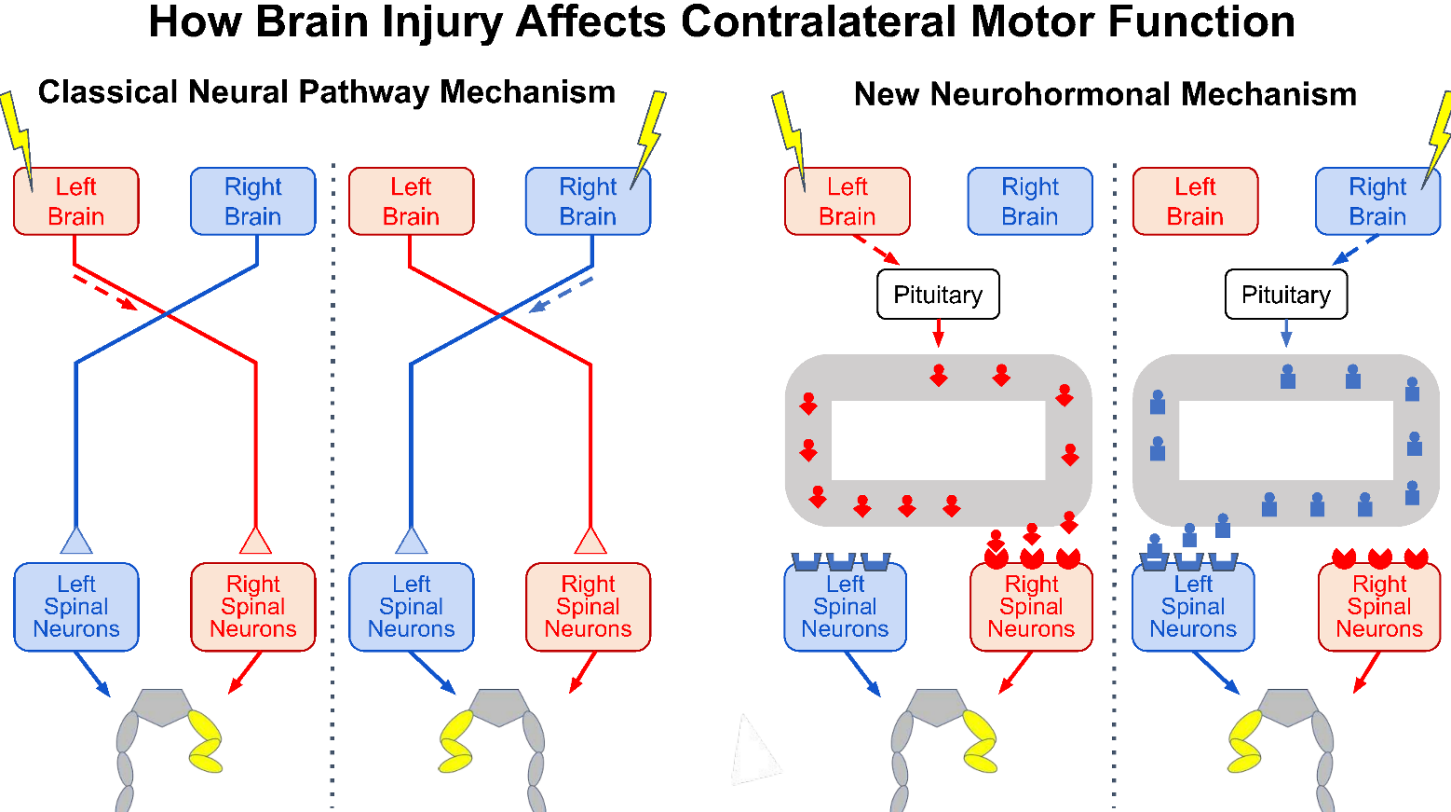
The effects of brain injury on contralateral motor function. Injury to one side of the brain (yellow lightning bolt) impairs motor function on the opposite side of the body, causing the abnormal flexed posture of the opposite hind leg (yellow). It now appears that two vastly different mechanisms can produce this effect. In the classical mechanism known for centuries (left panel), brain injury impairs the activity in neural pathways that cross to the other side of the spinal cord and synapse on spinal neurons controlling the opposite hind leg, thereby causing the abnormal hind-leg posture. In the new mechanism revealed by Lukoyanov et al. (right panel), brain injury stimulates the pituitary gland to secrete a side-specific neurohormone (red or blue icon) into the bloodstream (grey loop) that binds to receptors concentrated in spinal neurons on the opposite side, thereby also causing the abnormal hind-leg posture.

Now, in eLife, Georgy Bakalkin (Uppsala University), Jens Schouenborg (Lund University) and colleagues in Denmark, Portugal, Russia and Sweden – including Nikolay Lukoyanov, Hiroyuki Watanabe, Liliana Carvalho, Olga Nosova and Daniil Sarkisyan as joint first authors – report that neurohormones produced by the pituitary gland at the base of the brain may also contribute to the contralateral effect of brain injury (Lukoyanov et al., 2021). They build on previous studies showing that pituitary neurohormones can have side-specific effects in uninjured rats. If confirmed, these latest results are enormously significant, both scientifically and clinically.

In a straightforward set of experiments, the spinal cord of rats was completely transected in the mid-back, thereby cutting the neural pathways from the brain to the spinal neurons that control the hind-leg muscles on the opposite side. Then, the sensorimotor area of cerebral cortex was injured on one side of the brain. Surprisingly, the researchers observed an abnormal posture in the opposite hind leg similar to that seen when the sensorimotor cortex was injured in rats in which the spinal cord had not been transected.

To explore this further, the researchers removed the pituitary gland before transecting the spinal cord and injuring one side of the brain. With the pituitary removed and the spinal cord transected, the brain injury did not cause an abnormal posture in the opposite leg. Moreover, injecting healthy animals with specific pituitary hormones, or with an extract from the blood of brain-injured animals, produced a comparable abnormal posture on one side, thereby confirming a previously unrecognized contribution of neurohormones to the contralateral effect of a unilateral brain injury. This effect may result from the asymmetrical distribution of some neurohormonal receptors in the spinal cord (Figure 1, right side; Kononenko et al., 2017; Watanabe et al., 2021).

Perhaps the most remarkable aspect of these findings is that the phenomenon the researchers describe has gone largely unrecognized for so long. Certainly, the results require further studies, also involving other species. A thorough search of the clinical literature for reports about the effect of hemispheric stroke in people with complete spinal cord injuries would also be in order. But assuming that the results are confirmed and do occur in other species, in particular primates, the scientific questions they raise – and the clinical possibilities they introduce – are significant and exciting.

A variety of studies show that lateralized effects of neurohormones contribute to normal brain function (e.g., Deliagina et al., 2000; Marlin et al., 2015; Nation et al., 2018; Watanabe et al., 2015). It remains to be seen whether the lateralized effects of neurohormones following brain injury are limited to motor function or, more plausibly, whether they also affect other nervous system functions, such as vision or language. Language problems often occur with stroke on the left side of the brain, and neurohormones might play a role in these difficulties.

It is also unclear how lateralized neurohormonal effects interact with lateralized neural pathway effects. They clearly differ in mechanisms, and probably in other respects as well, such as time frames. Are their interactions synergistic, additive or opposing, or does this vary from one situation to another? Another important question is how the lateralized effects of neural pathways and neurohormones operate in normal life. How do they interact to ensure that normal function is maintained throughout life? For example, learning new motor skills is currently studied and understood entirely in terms of neuronal and synaptic plasticity in neural pathways (e.g., Dayan and Cohen, 2011). Do neurohormones contribute as well?

The work of Lukoyanov et al. adds a whole new dimension to the problem of brain injury and to the opportunities for new therapies that enhance recovery. They studied neurohormonal effects in the first few hours after injury; thus, the long-term effects are unknown. For example, neurohormones might contribute to the abnormal movement patterns that emerge after stroke (e.g., Senesh et al., 2020). Knowledge of their acute and chronic effects could help shape the design of new therapeutic regimens, both in general and for individual patients; appropriate treatments may well differ depending on which side of the brain is injured. Furthermore, new classes of therapeutic agents that mimic or oppose neurohormones, or affect their endogenous production, might enhance recovery of useful function beyond that achievable with present methods.

In summary, the truly groundbreaking research of Lukoyanov et al. opens a new research area and demands a host of further studies. If the results are replicated, the impact, excitement and activities they will generate are likely to continue growing well into the future.
